# Polyglutamine-Expanded Ataxin-3 Accelerates CFTR Degradation Through K63-Linked Ubiquitination to Exacerbate Microglial Inflammation

**DOI:** 10.1080/17590914.2026.2662867

**Published:** 2026-05-26

**Authors:** Zixin Wang, Xue Fan, Bingbing Bai, Mengxue Song, Ye Shi, Yinghao Zhao, Pengyu Xie, Tao Shi, Chunjiu He, Yuhong Hu, Qingtian Wu, Xia Hou

**Affiliations:** a Department of Genetics, Jiamusi University School of Basic Medicine, Jiamusi, Heilongjiang, China; b Key Laboratory of Microecology-Immune Regulatory Network and Related Diseases, Jiamusi University, Jiamusi, Heilongjiang, China; c Department of Pathogeny Biology, Jiamusi University School of Basic Medicine, Jiamusi, Heilongjiang, China; d Department of Respiratory, The First Affiliated Hospital of Jiamusi University, Jiamusi, Heilongjiang, China; e Department of Anesthesiology, The First Affiliated Hospital of Jiamusi University, Jiamusi, Heilongjiang, China; f Department of Gynecology and obstetrics, The First Affiliated Hospital of Jiamusi University, Jiamusi, Heilongjiang, China

**Keywords:** Ataxin-3, cystic fibrosis transmembrane transduction regulator CFTR, deubiquitinase, microglia, neurodegenerative diseases, neuroinflammation

## Abstract

The deubiquitinase Ataxin-3 causes spinocerebellar ataxia type 3 (SCA3) upon polyglutamine (polyQ) expansion. While expressed in the nervous system, the function of the cystic fibrosis transmembrane conductance regulator (CFTR) chloride channel therein remains unclear, as does its potential regulation by Ataxin-3. This study reveals that Ataxin-3 interacts with and promotes CFTR degradation in human microglia by its K63-linked polyubiquitination, thereby shortening CFTR’s half-life. Paradoxically, K63-linked polyubiquitin chains also promote the degradation of Ataxin-3 itself, suggesting a complex feedback mechanism. The pathogenic Ataxin-3Q80 mutant exerts a stronger effect than the wild-type protein. Consequently, this Ataxin-3–CFTR axis drives microglial polarization toward a pro-inflammatory phenotype and amplifies neuroinflammation. We thus identify a novel “Ataxin-3–K63 ubiquitin chain–CFTR” pathway that controls microglial activation, offering new mechanistic insight and therapeutic targets for SCA3. **Abbreviations:** MJDM: achado-Joseph disease; SCA3: spinocerebellar ataxia type 3; PolyQ: polyglutamine; CNS: central nervous system; CFTR: cystic fibrosis transmembrane conductance regulator; CF: cystic fibrosis; UIMs: ubiquitin-interacting motifs; MEM: Minimum Essential Medium; FBS: fetal bovine serum; P/S: penicillin/streptomycin; siRNA: small interfering RNA; BSA: bovine serum albumin; Co-IP: Co-immunoprecipitation; LPS: lipopolysaccharide; WT-CFTR: wild-type CFTR; CHX: Cycloheximide; 3-MA: 3-Methyladenine; IF: Immunofluorescence; IB: immunoblot; Ub: ubiquitin

## Background

Machado-Joseph disease (MJD), also known as spinocerebellar ataxia type 3 (SCA3), is a rare, debilitating, and incurable hereditary neurodegenerative disorder. Its clinical manifestations induce cerebellar ataxia, progressive loss of brain and spinal cord volume as well as motor dysfunction, local shape abnormalities of subcortical structures, progressive external ophthalmoplegia, dystonia, and neuroinflammatory responses. These symptoms worsen with disease progression, ultimately leading to severe motor impairment and diminished quality of life (Conceição & Manuela, 2011; Costa & Paulson, 2012; Potapenko et al., 2024; Rüb et al., 2013; Udo et al., 2008; Ye et al., 2025; Yuan et al., 2025). Genetically, SCA3 is caused by an abnormal expansion of the CAG trinucleotide repeat within the *ATXN3* gene, which encodes a polyglutamine (polyQ) tract within the Ataxin-3 protein (Raj & Akundi, 2021).

Ataxin-3, is a deubiquitinating enzyme expressed in microglia, the resident macrophages of the central nervous system (CNS), and plays crucial roles in the ubiquitin-proteasome system and transcriptional regulation (Evers et al., 2014). Microglia contribute to neurodegenerative pathogenesis by driving pro-inflammatory immune responses (Borst et al., 2021; Rodríguez-Gómez et al., 2020). Microglia cells undergo phenotypic polarization into pro-inflammatory or anti-inflammatory states, which are key regulatory points in neuroinflammation. M1 polarization promotes the release of pro-inflammatory cytokines, exacerbating neuronal damage, whereas M2 polarization exerts neuroprotective effects through anti-inflammatory factors (Shenrui et al., 2022). Despite this, the precise mechanism by which polyQ-expanded Ataxin-3 drives microglia-mediated neuroinflammation in SCA3 remains incompletely understood.

Interestingly, the cystic fibrosis transmembrane conductance regulator (CFTR), a chloride channel protein best known for its role in cystic fibrosis (CF), has recently emerged as a modulator of immune and inflammatory responses (Keown et al., 2020). Its dysfunction has been linked to altered microglial activity, suggesting a potential, though unexplored, connection to SCA3 pathology. The stability of CFTR is regulated by ubiquitination, which targets it for degradation via both the proteasomal and lysosomal pathways (Jensen et al., 1995; Lukacs et al., 1994; Ward et al., 1995; Ward & Kopito, 1994). This ubiquitin-mediated regulation presents a potential link to Ataxin-3. Ataxin-3 contains ubiquitin-interacting motifs (UIMs) that bind polyubiquitin chains and facilitate substrate processing (Berke et al., 2005; Nicastro et al., 2010; Song et al., 2010). Notably, Ataxin-3 can bind both K48- and K63-linked polyubiquitin chains and has been shown to preferentially cleave K63-linked chains *in vitro* (Evers et al., 2014). This specific deubiquitinase activity suggests that Ataxin-3 could modulate the stability of substrate proteins like CFTR by editing its ubiquitin chains. However, the function relationship between Ataxin-3 and CFTR has not been investigated.

In this study, we demonstrate that Ataxin-3 promotes degradation of CFTR, particularly the form modified with K63-linked polyubiquitin chains. Paradoxically, K63-linked polyubiquitin chains also promote the degradation of Ataxin-3 itself. We further show that polyQ-expanded mutant Ataxin-3Q80 exhibits a stronger effect than the wild-type protein in driving CFTR degradation and polarizing microglia towards the pro-inflammatory phenotype, thereby enhancing the secretion of inflammatory cytokines. Collectively, our findings delineate a novel “Ataxin-3–K63 ubiquitin chain–CFTR” axis that regulates microglial polarization and neuroinflammation. This discovery provides a novel perspective on the pathogenesis of SCA3 and identifies potential therapeutic targets for this neurological disorder.

## Methods

### Cell Culture and Transfection

The human microglial cell line HMC-3 was purchased from Saibaikang (Shanghai, China) maintained in Minimum Essential Medium (MEM) medium supplemented with 10% fetal bovine serum (FBS) and 1% penicillin/streptomycin (P/S). The following plasmids were utilized: CFTR, overexpressed Ataxin-3 (Ataxin-3Q22), the polyQ-expanded mutant (Ataxin-3Q80), and the corresponding catalytically inactive polyQ-expanded mutant (Ataxin-3C14A), ubiquitin and ubiquitin lysine mutant we reported (Wu et al., 2022). Transfections were performed using Lipofectamine 2000 (Invitrogen) and Dorner transfection reagents, according to the manufacturers’ protocols for siRNA or DNA plasmid. Small interfering RNAs (SiRNAs) were synthesized by Sangon Biotech (Shanghai, China), and their sequences are listed in Supplementary Table 1.

### Antibodies

The following primary antibodies were used: mouse monoclonal and rabbit monoclonal anti-CFTR, and mouse monoclonal anti-Ataxin-3 (all from Proteintech Group, Inc). Anti-hemagglutinin (HA) rabbit polyclonal, anti-GAPDH mouse monoclonal, anti-K48-ubiquitin, and anti-K63-ubiquitin rabbit polyclonal antibodies were obtained from ABclonal. All primary antibodies were diluted in a commercial antibody diluent (Beyotime Biotechnology) prior to Western blot analysis.

### Immunofluorescence Staining

HMC-3 cells were seeded on glass-bottom dishes and transfected at 70%–80% confluency. Forty-eight hours post-transfection, cells were fixed with 4% paraformaldehyde for 20 minutes, permeabilizated with 0.5% Triton X-100 for 20 minutes, and then was blocked with 5% bovine serum albumin (BSA) in TBST for one hour, cells were then incubated overnight at 4 °C with primary antibodies against CFTR (rabbit monoclonal, 1:500) and Ataxin-3 (mouse monoclonal, 1:1,000) in antibody diluent. Subsequently, cells were incubated with Alexa-488-conjugated goat anti-mouse and Cy3-conjugated goat anti-rabbit secondary antibodies for one hour at room temperature in the dark. Nuclei were stained with DAPI, and images were captured using a confocal laser scanning microscope.

### Proximity Ligation Assay

When HMC-3 cells reach 80%–100% confluency and cultured for 48 hours. The medium was discarded, and cells were washed with PBS. Fixation was performed using 4% paraformaldehyde for 20 minutes, followed by permeabilization with 0.5% Triton X-100 in 4% paraformaldehyde for 20 minutes at RT. Samples were blocked with 5% BSA in TBST for one hour at RT, then incubated overnight at 4 °C with the primary antibodies against Atranxin-3 and CFTR to allow binding between the primary antibody and the target protein. After three washed with a washing buffer, corresponding PLA probes are added and incubated for one hour at RT. Ligase and ligation oligonucleotides were added, and the ligation reaction was carried out at a 37 °C for 30 minutes. DNA polymerase and the reaction buffer in TBST were then added for rolling-circle amplification 37 °C for 90 minutes. Labeled oligonucleotides were added to hybridize with the complementary sequences within the amplicons and signals were detected using fluorescence microscope.

### Western Blotting

Cell lysis buffer was prepared by mixing NP-40 Lysis Buffer (Cat. #P0013F, Beyotime Biotechnology Inc., China) with protease inhibitor cocktail (Cat. #K1007, APExBIO Technology LLC, USA) at a ratio of 100:1. The protein concentration of the collected supernatant was determined using the BSA assay, with absorbance measured at a wavelength of 540 nm on a BioTek multifunctional microplate reader.

A total of 40 μg of protein lysate was loaded onto 8% polyacrylamide gels for electrophoretic separation. Following electrophoresis, proteins were transferred onto polyvinylidene difluoride (PVDF) membranes (Cat. #FFP32, Beyotime Biotechnology Inc., China). The membranes were blocked with 5% non-fat milk (Yili Group, China) dissolved in 1× TBS-Tween20 buffer for 1 hour at room temperature (RT), and then incubated with primary antibodies for 2 hours at RT. The primary antibodies used were as follows: CFTR (Cat. #66928-1-Ig, 1:5000, Sanying Biotechnology Inc., Wuhan, China; Cat. #A8386, 1:1000, Abclonal Technology Co., Ltd., China) Ataxin-3 (Cat. #67057-1-Ig, 1:20000, Sanying Biotechnology Inc., Wuhan, China) GAPDH (Cat. #AC033, 1:25000, Abclonal Technology Co., Ltd., China) HA (Cat. #AE036, 1:2000, Abclonal Technology Co., Ltd., China). After primary antibody incubation, the membranes were washed three times with 1× TBST buffer for 10 minutes per wash. Subsequently, the membranes were incubated with horseradish peroxidase (HRP)-conjugated secondary antibodies (Cat. #A0192; Cat. #A0352, 1:2000, Beyotime Biotechnology Inc., China) for 1 hour at RT, followed by another three washes with 1× TBST buffer (10 minutes per wash). Protein bands were visualized using ECL chemiluminescent substrate (Cat. #MA0186-2, MBL International Co., Ltd., Dalian, China), and images were captured with a Tanon automatic chemiluminescence imaging system (Tanon Science & Technology Co., Ltd., Shanghai, China). Grayscale analysis of protein bands was performed using ImageJ software, with the implementation of a two-step normalization method: first, normalizing the grayscale values of target protein bands to the internal reference protein (GAPDH); second, calibrating the normalized values against the mean value of the control group.

### Co-Immunoprecipitation and Protein Ubiquitination Assays

To detect the endogenous interaction between Ataxin-3 and CFTR, co-immunoprecipitation (Co-IP) was performed on HMC-3 cells grown in 10-cm dishes. First, the cell lysis buffer was prepared by mixing NP-40 Lysis Buffer (Cat. #P0013F, Beyotime Biotechnology Inc., China) with protease inhibitor cocktail (Cat. #K1007, APExBIO Technology LLC, USA) at a ratio of 100:1. The cells were lysed on ice for 30 minutes, followed by centrifugation to collect the supernatant. Protein A + G Agarose (Cat. #P2012, Beyotime Biotechnology Inc., China) was washed three times with pre-chilled PBS buffer (Cat. #G4202-100ML, Save Biotechnology Co., Ltd., China). The prepared cell lysate was then added to the washed agarose beads and incubated at 4 °C for 1 hour to eliminate non-specific binding. After this pre-clearing step, the cell lysate was incubated with the corresponding primary antibody at 4 °C overnight. Following incubation, the agarose beads were washed five times with PBS buffer. The immunoprecipitated complexes were resuspended in 2× SDS-PAGE loading buffer, incubated at 42 °C for 10 minutes, and then boiled at 100 °C for 5 minutes. Finally, the samples were subjected to western blot analysis for detection.

For ubiquitination assay, HMC-3 cells were co-transfected with plasmids encoding Ataxin-3 or its mutants, HA-tagged Ubiquitin. Approximately 24 hours post-transfection. Cells were treated with 20 µM MG-132 for 4 hours before harvesting according to experiments request. Cell lysates were immunoprecipitated with an anti-CFTR antibody, followed by immunoblotting with an anti-HA antibody.

### Cell Viability Assay

HMC-3 cells were seeded at 1,000 cells per well in 96-well plates. After 24 hours, the culture medium was replaced with fresh medium containing lipopolysaccharide (LPS) at various concentrations (serially diluted from a 1 mg/mL stock solution) and incubated for different durations. Following the addition of 10% CCK-8 reagent, the samples were incubated for 2 hours, and absorbance was measured at 450 nm.

### Cycloheximide (CHX) Chase

Approximately 40 hours after transfection, HMC-3 cells were treated with 100 μg/mL CHX about four hours, and then harvested at indicated time points to prepare cell lysate for Western blot analysis.

### Quantitative Real-Time PCR

Total RNA was extracted using Trizol reagent (Invitrogen) and reverse transcribed using BeyoRT^TM^ II cDNA synthesis kit (with gDNA Eraser). Quantitative real-time PCR was performed on a 7300 Real Time PCR System machine using GS AntiQ qPCR SYBR Green Fast Mix (Universal). All primers used were purchased from Sangon Bioengineering and primer sequences are shown in Supplementary Table 2.

### Protein Staining on Gel

Coomassie Brilliant Blue Staining. Following SDS-PAGE gradient gel separation, the gel was placed in 0.05% Coomassie Brilliant Blue staining solution (40% ethanol, 10% glacial acetic acid, 50% ddH_2_O) at 60°Cfor one hour in with gentle shaking. The gel was then destained with destaining solution (40% ethanol, 10% glacial acetic acid) at room temperature with gentle agitation until the background becomes clear.

SYPRO Ruby Staining. The gradient gel was fixed twice with fix solution (50% methanol, 7% acetic acid) for 15 minutes with gentle agitation. The gel was then incubated overnight in the dark with SYPRO^®^ Ruby Protein Gel Stain (Thermofisher Scientific) with gentle agitation. Subsequently, the gel was washed in washing solution (10% methanol, 7% acetic acid), followed by two rinses in ultrapure water (5 minutes per rinse). The stained gel was visualized under UV light.

### LC-MS/MS Analysis

Liquid chromatography-tandem mass spectrometry (LC-MS/MS) analysis was performed as previously described (Hou et al., 2019).

### Statistical Analysis

All experiments were performed in at least three independent replicates. Data are presented as mean ± SD, and differences between groups were analyzed by one-way ANOVA. A p-value of less than 0.05 was considered statistically significant. All statistical analyses were conducted using GraphPad Prism 8.0.2 software. All experiments were repeated three times, with representative results.

## Results

### Ataxin-3 Interacts with CFTR in Microglia Cell HMC-3

**Figure 1.**
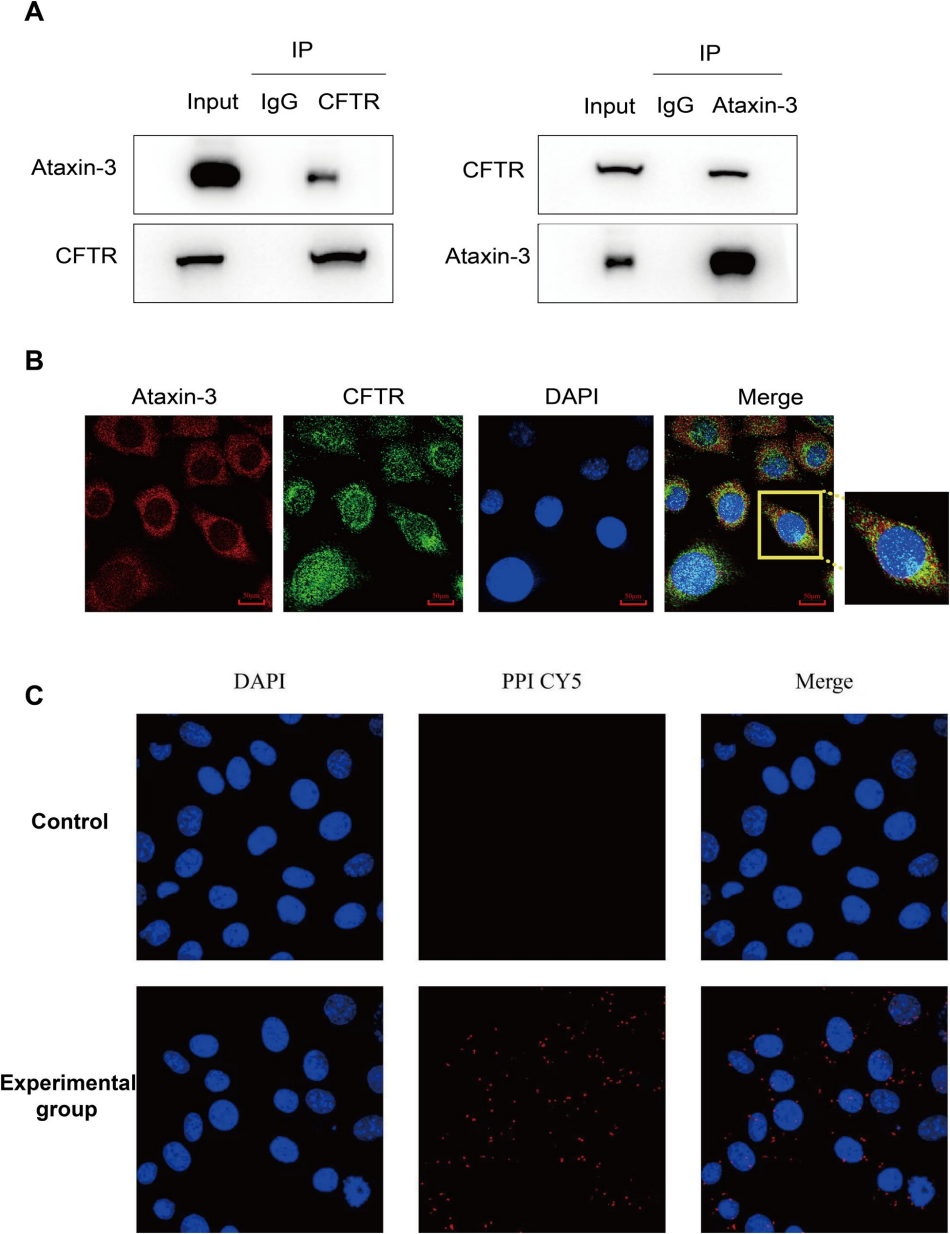
Ataxin-3 Endogenously interacts and co-localizes with CFTR in microglia HMC-3 cell. (A) Endogenous interaction between Ataxin-3 and CFTR was assessed by Co-IP in HMC-3 cell lysates. Proteins were immunoprecipitated using an anti-CFTR antibody or an anti-Ataxin-3 antibody, with normal IgG serving as a negative control. Immunoprecipitates were immunoblotted (IB) with the indicated antibodies. Positive controls: CFTR IP with CFTR antibody and Ataxin-3 IP with Ataxin-3 antibody. (B) Endogenous co-localization of Ataxin-3 and CFTR in HMC-3 cells. Cells were subjected to immunofluorescence staining for Ataxin-3 (red) and CFTR (green). Nuclei were counterstained with DAPI (blue). Scale bar, 50 μm (C) Proximity Ligation Assay (PLA) was used to verify the interaction between Ataxin-3 and CFTR in HMC-3 cells. Heterologous antibodies against Ataxin-3 and CFTR were selected, and the corresponding PLA probes were added to form a closed circular DNA. Rolling-circle amplification was performed using the PLA probes as primers and the circular DNA as the template to synthesize concatenated sequences. The experimental group was incubated with both anti-Ataxin-3 antibody and anti-CFTR antibody, while the control group was incubated with IgG antibody. The number and distribution of PLA signals were observed. Nuclei were counterstained with DAPI (blue). Scale bar, 50 μm. IP, IF, and PLA assays were performed with three independent biological replicates (n = 3).

Since Ataxin-3 and CFTR are expressed in microglia and implicated in inflammation, we hypothesized that Ataxin-3 might contribute to MJD pathogenesis by regulating CFTR. We first investigated whether Ataxin-3 interacts with CFTR in the human microglial cell line HMC-3. MJD is primarily caused by CAG repeat expansion in the *ATXN3* gene, leading to protein misfolding, aggregation, and inclusion body formation. Microglia can modulate neurodegeneration by clearing misfolded proteins via phagocytosis or by releasing inflammatory mediators that exacerbate aggregation and neuronal damage (Colonna & Butovsky, 2017). Confirming their expression in microglia in The Human Protein Atlas database (unicellular type - Ataxin-3 - The Human Protein Atlas; unicellular type - CFTR - The Human Protein Atlas. CFTR is expressed in human microglia at 24.1 nCPM [normalized counts per million], representing a low but readily detectable expression level. Ataxin-3 is expressed in human microglia at 82.6 nCPM [normalized counts per million], corresponding to a moderate expression level). we validated an endogenous interaction by immunoprecipitating CFTR from cell lysates of HMC-3 (Figure 1A). To further corroborate this binding, we performed Co-IP in human HEB cell line CFBE-41o-WTCFTR stably expressing wild-type CFTR, using HA antibody and IgG as the negative control (Supplementary Figure 1A). Then immunoprecipitates were separated by SDS-PAGE and stained by Coomassie brilliant blue R250 (Supplementary Figure 1B) or SYPRO Ruby (Supplementary Figure 1C). A band (in blue box) matching the size of purified Ataxin-3 protein (in yellow box, a gift from Dr. Sokol V. Todi (Blount et al., 2014)) was excised for mass spectrometry, which unequivocally identified the protein as Ataxin-3 (Supplementary Figure 1D). Furthermore, immunofluorescence staining confirmed the colocalization of Ataxin-3 and CFTR in the cytoplasm of HMC-3 cells (Figure 1B). To confirm the interaction between the two proteins, we performed a proximity ligation assay (PLA). By observing and recording the number and distribution of PLA signals, we determined that Ataxin-3 and CFTR interact with each other (Figure 1C). The presence of PLA signals (red dots) in HMC-3 cells incubated with both anti-Ataxin-3 and anti-CFTR antibodies, but not in control cells (incubated with IgG), demonstrated a physical interaction between Ataxin-3 and CFTR (Figure 1C). Collectively, these results demonstrate that Ataxin-3 and CFTR endogenously and interact and co-localize in human microglial cells. But our data do not rule out the involvement of bridging molecules, including other proteins, RNA, or DNA.

### The Ataxin-3 Mutant PolyQ-Expanded Ataxin-3Q80 Promotes CFTR Degradation via Dual Pathways

**Figure 2.**
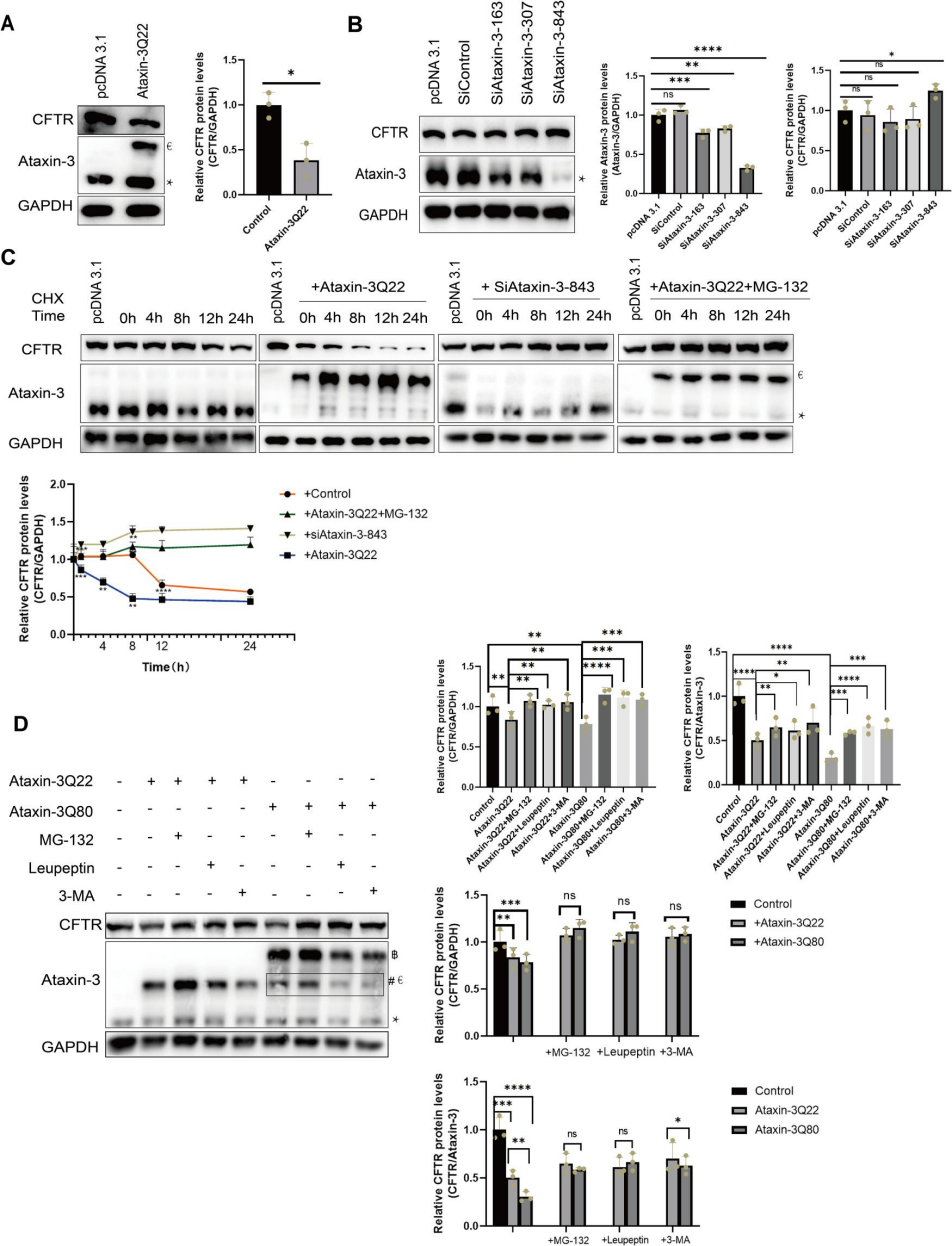
Overexpressed Ataxin-3 and polyQ-expanded mutant Ataxin-3Q80 promote CFTR degradation. (A) Overexpression of Ataxin-3 Enhances CFTR Protein Degradation. overexpressed Ataxin-3 (Ataxin-3Q22) was transfected into HMC-3 cells for 48 hours. Cell lysates were analyzed by Western blotting. *represents the endogenous Ataxin-3 band, €represents overexpressed Ataxin-3. Data are presented as mean ± SD. The significance of differences in mean values between the two groups was analyzed using Student’s *t*-test. All experiments were performed with 3 independent biological replicates (n = 3), *p < 0.05. (B) Endogenous Ataxin-3 Knockdown Stabilizes CFTR Protein Expression. Three specific Ataxin-3 siRNAs and a non-targeting siRNA control (si-control) were transfected into HMC-3 cells for 48 hours. *represents the endogenous Ataxin-3 band. The data are presented as the mean ± SD. One-way analysis of variance (ANOVA) followed by Dunnett’s multiple comparison test revealed a statistically significant overall difference in Ataxin-3 and CFTR protein expression levels among the experimental groups. Experiments were independently repeated three times (n = 3). ns, not significant, *p < 0.05, **p < 0.01, ***p < 0.001, ****p < 0.0001. (C) Overexpression of Ataxin-3 Shortens CFTR Half-Life. The control group was left untreated, while the experimental groups were transfected with Ataxin-3Q22 and Ataxin-3 siRNA-843 in HMC-3 cells for 48 hours, and then treated with 20 μM MG-132 for 4 hours the next day. Cells were treated with 100 μg/mL Cycloheximide and lysates were collected at appointed time points for Western blot analysis. CFTR protein levels were normalized and shown on the right panel. *represents the endogenous Ataxin-3 band, €represents overexpressed Ataxin-3. Data are presented as mean ± SD, one-way ANOVA indicated overall significant differences in the expression of CFTR proteins among the experimental groups. Experiments were independently repeated three times (n = 3). *p < 0.05, **p < 0.01, ***p < 0.001, ****p < 0.0001. (D) Ataxin-3 modulates CFTR protein levels, at least in part, through proteasomal and lysosomal degradation pathways. Ataxin-3Q22 and Ataxin-3Q80 were transfected into HMC-3 cells. On the first day post-transfection, cells were treated with 2 μg/mL Leupeptin for 24 hours. On the second day, cells were subsequently treated with 10 mM 3-MA or 20 μM MG-132 for 12 hours or 4 hours, respectively. Cell lysates were then collected for Western blot analysis. *represents the endogenous Ataxin-3 band, €represents overexpressed Ataxin-3, ฿ represents mutant Ataxin-3, # indicates a non-specific band. The statistical analysis was performed on the expression levels of CFTR protein in cells overexpressing wild-type or expanded Ataxin-3. Data are presented as mean ± SD. The data were normalized to GAPDH and Ataxin-3, respectively, and statistical analysis was performed using one-way analysis of variance (ANOVA) followed by Dunnett’s multiple comparison test. All experiments were conducted with three independent biological replicates. *p < 0.05, **p < 0.01, ***p < 0.001, ****p < 0.0001.

After confirming the interaction, we explored the functional consequence of Ataxin-3 on CFTR protein stability. Transient overexpression of Ataxin-3Q22 in HMC-3 cells significantly reduced CFTR protein levels to approximately 45% compared to pcDNA3.1 vector control (Figure 2A). To confirm this, we knocked down endogenous Ataxin-3 using three specific siRNAs (siRNA-163, siRNA-307, and siRNA-843). SiRNA-843, the most efficient one, reducing Ataxin-3 to 33% (Figure 2B), was used in subsequent experiments.

Given CFTR has a reported half-life of 12–24 hours (Figure 2C) (Cheng et al., 1990), we performed CHX-chase assays to determine if Ataxin-3 alters this. Overexpression of Ataxin-3Q22 shortened the CFTR half-life to approximately 4 hours (CHX concentration shown in Supplementary Figure 2A). Conversely, CFTR degradation was markedly delayed upon Ataxin-3 knockdown or treatment with proteasome inhibitor MG-132, with CFTR remaining detectable beyond 24 hours (Figure 2C). Notably, MG-132 blocked Ataxin-3-mediated CFTR degradation, indicating a partial dependence on the proteasome pathway (Figure 2C, Supplementary Figure 2B).

Overexpressed Ataxin-3 contains a polyQ tract (typically 10 to 41 repeats), with expansions beyond 51 repeats causing SCA3/MJD. To test if this expansion influences its ruining role on CFTR, we transfected HMC-3 cells with overexpressed Ataxin-3Q22 or the mutant Ataxin-3Q80 (80 repeats). Treatment with proteasome inhibitor (MG-132), lysosomal inhibitor (leupeptin), or autophagy inhibitor (3-MA) revealed that both Ataxin-3Q22 and Ataxin-3Q80 mutant caused a significant reduction in CFTR protein levels. After normalization against the corresponding GAPDH protein expression levels and subsequent statistical analysis, it was found that there was no significant difference in the extent of CFTR protein reduction induced by the Ataxin-3Q22 and Ataxin-3Q80 mutants. However, this statistical method could not account for the influence exerted by Ataxin-3 itself, representing a limitation. Following normalization to the expression levels of the corresponding Ataxin-3 protein and subsequent statistical analysis, the Ataxin-3Q80 mutant was found to induce a significantly greater decrease in CFTR protein levels compared with Ataxin-3Q22 (a reduction of ∼55% *vs.* ∼38%), suggesting that polyglutamine tract expansion enhances the degradative activity of Ataxin-3. When overexpressing overexpressed Ataxin-3Q22, this degradation was attenuated by both proteasome and autophagy inhibitors; whereas with mutant Ataxin-3Q80 overexpression, the degradation was mediated through both proteasomal and lysosomal pathways. This indicates that both wild-type and mutant Ataxin-3 promote CFTR degradation via dual degradation pathways (Figure 2D).

### K63-Linked Polyubiquitin Chain Downregulate Ataxin-3 and Partially Rescues CFTR from Degradation

**Figure 3.**
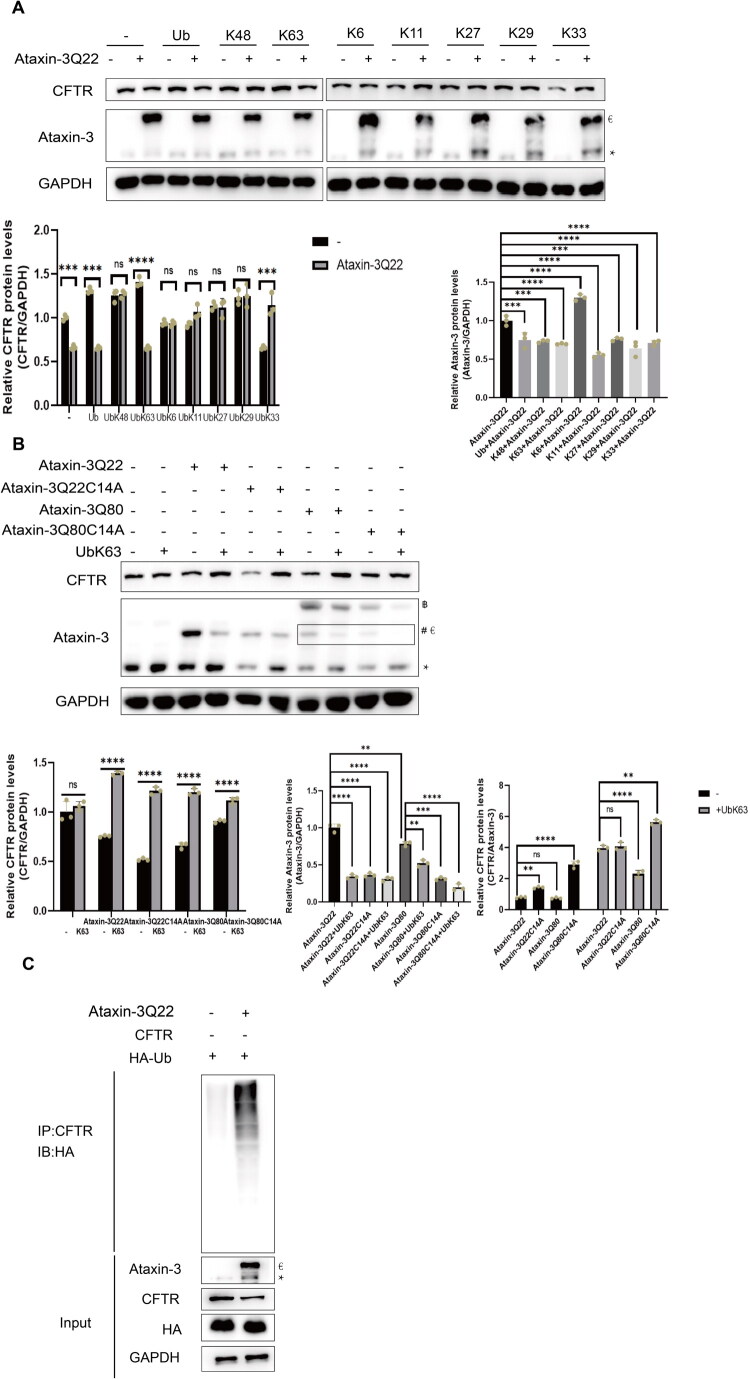
K63 linked poly-ubiquitin chains downregulates Ataxin-3 and partially rescue CFTR. (A) Ataxin-3 ruined CFTR rescued by K63 linked poly-ubiquitin chains. HMC-3 cells were co-transfected with Ataxin-3Q22 and the indicated ubiquitin (Ub) plasmids for 48 hours. CFTR protein level was analyzed by Western blot. Quantification of normalized CFTR levels are shown on the right panel. *represents the endogenous Ataxin-3 band, €represents overexpressed Ataxin-3. The plasmid maps for all ubiquitin constructs shown in this figure (UB, Ub-K6, Ub-K11, Ub-K27, Ub-K29, Ub-K33, Ub-K48, and Ub-K63) are available in Supplementary Figure 3A. The data are presented as the mean ± SD. One-way analysis of variance (ANOVA) followed by Dunnett’s multiple comparison test revealed a statistically significant overall difference in Ataxin-3 and CFTR protein expression levels among the experimental groups. Experiments were independently repeated three times (n = 3). ns, not significant, ***p < 0.001, ****p < 0.0001. (B) The degradative function of Ataxin-3 is enhanced by polyQ expansion and rescued by Ub-K63. HMC-3 cells were co-transfected with Ub-K63 and the indicated Ataxin-3 constructs (WTQ22, Q80, Q22C14A, Q80C14A) for 48 hours. CFTR protein levels were analyzed by Western blot (left). Quantification of normalized CFTR and Ataxin-3 levels are shown on the lower panels. * represents the endogenous Ataxin-3 band, €represents overexpressed Ataxin-3, ฿ represents mutant Ataxin-3. The data are presented as the mean ± SD. One-way analysis of variance (ANOVA) followed by Dunnett’s multiple comparison test revealed a statistically significant overall difference in Ataxin-3 and CFTR protein expression levels among the experimental groups. Experiments were independently repeated three times (n = 3). ns, not significant, **p < 0.01, ***p < 0.001, ****p < 0.0001. (C) Ataxin-3 enhances CFTR polyubiquitination. HMC-3 cells were co-transfected with CFTR, HA-Ub, and Ataxin-3Q22 as indicated for 48 hours. Cell lysates were subjected to immunoprecipitation with an anti-CFTR antibody, followed by immunoblotting with an anti-HA antibody to detect ubiquitinated CFTR. * represents the endogenous Ataxin-3 band, €represents overexpressed Ataxin-3. All experiments were performed in triplicate (n = 3).

Ataxin-3 is a deubiquitinase that binds to K48 and K63-linked chains but preferentially cleaves K63-linked polyubiquitin (Matos et al., 2016). Since ubiquitination targets CFTR for degradation, we investigated the linkage type probably involved in Ataxin-3-mediated regulation. We used a panel of HA-tagged ubiquitin mutants, each retaining only one lysine (K) residue (K6, K11, K27, K29, K33, K48, and K63) (Supplementary Figure 3A). Co-transfection into HMC-3 cells showed that CFTR modified by K63-linked chains increased significantly, and this modification was reduced by Ataxin-3Q22 (Figure 3A). Consistent with this, Ataxin-3Q22 was downregulated by wild-type Ub and Ub-K63 in HMC-3 cells, but not in HEK293 cells (Figure 3A and Supplementary Figure 3B), suggesting a cell type-specific regulation and a key role for K63 chains.

To confirm this, we co-transfected Ub-K63 with Ataxin-3Q22 or its mutants (Q80, C14A, Q80/C14A) into HMC-3 cells. The catalytic activity of Ataxin-3 depends on a triad (C14, H119, N134) in its Josephin domain, with the C14A mutation ablating deubiquitinase activity (Burnett et al., 2003; Ginhoux et al., 2010; Mao et al., 2005). Both Ataxin-3Q22 and Ataxin-3Q80 significantly decreased CFTR levels, with the Q80 mutant showing stronger activity. Ub-K63 partially rescued CFTR from degradation induced by all constructs. Surprisingly, the catalytically inactive C14A mutant exhibited enhanced, not reduced, ability to promote CFTR degradation (Figure 3B), an effect also observed in HEK293 cells (Supplementary Figure 3B–D). Furthermore, Ub-K63 markedly reduced the protein levels of Ataxin-3Q22 and its mutants in HMC-3 cells, but not in HEK293 cells (Figure 3A and B, Supplementary Figure 3B–D). Quantification of Ataxin-3 levels in HEK293 cells confirmed no significant change upon Ub-K63 overexpression, validating the cell type-specific phenomenon (Supplementary Figure 3D). These data elucidate that the polyQ expansion (Q80) and catalytic inactivation (C14A) both enhance Ataxin-3’s degradative function, and that Ub-K63 can partially protect CFTR from this degradation.

Finally, a ubiquitination assay confirmed that Ataxin-3 significantly enhances the polyubiquitination of CFTR in HMC-3 cells (Figure 3C). Our results suggest that Ataxin-3 facilitates CFTR degradation, likely through mediating the conjugation of polyubiquitin chains to CFTR.

### Ataxin-3 Promotes Microglial Polarization Shift toward the Pro-Inflammatory Phenotype under LPS Stimulation

**Figure 4.**
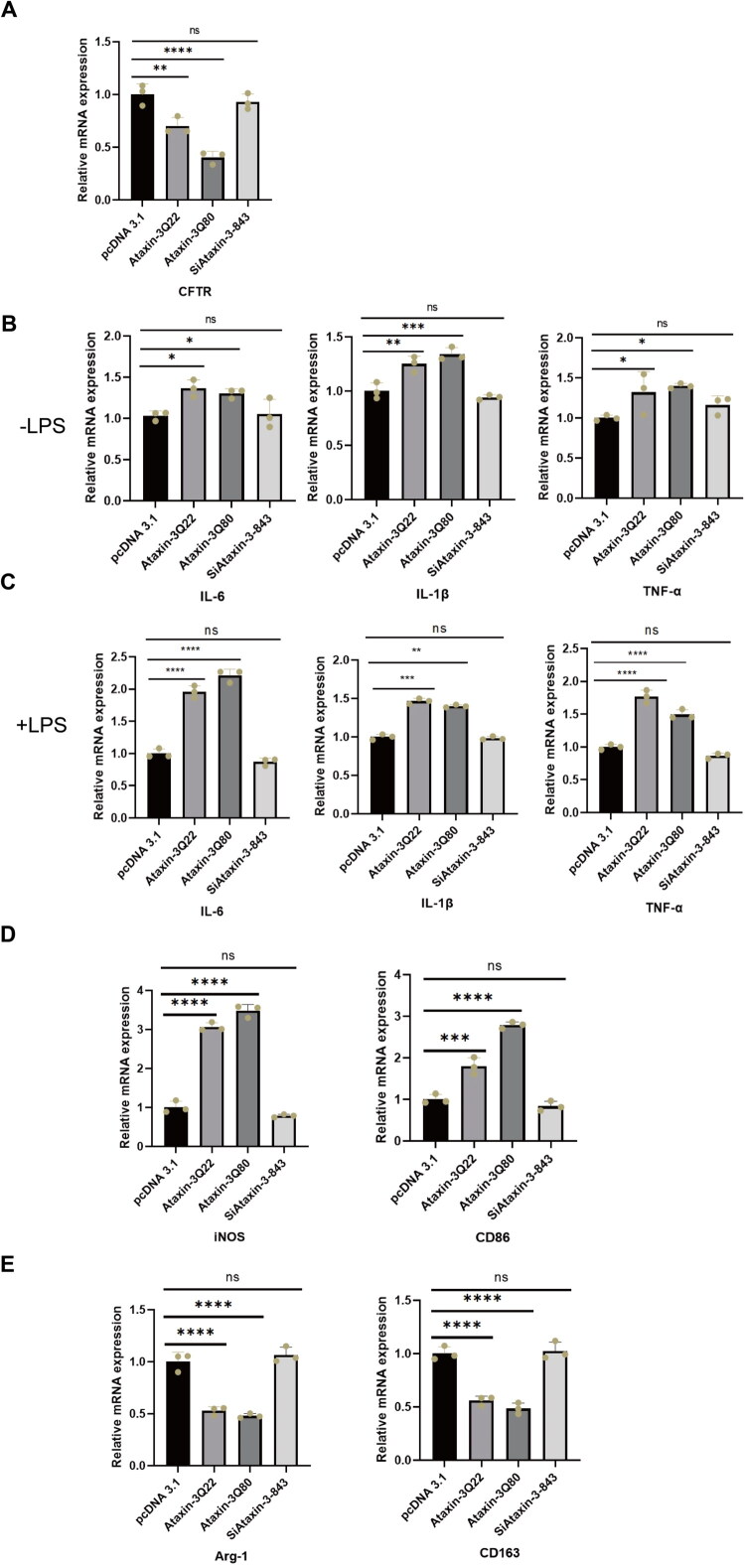
Ataxin-3 induces an inflammatory response and promotes a shift toward the M1 phenotype in HMC-3 cells. HMC-3 cells were transfected with overexpressed Ataxin-3 (Q22), mutant Ataxin-3Q80, or si-Ataxin-3-843 as indicated. mRNA levels were measured by qRT-PCR. (A) mRNA levels of the CFTR. (B) mRNA levels of the pro-inflammatory cytokines IL-1β, IL-6, and TNF-α without LPS stimulation. (C) mRNA levels of the pro-inflammatory cytokines IL-1β, IL-6, and TNF-α under stimulation with 200 ng/mL LPS for 4 hours. (D) mRNA levels of M1 (pro-inflammatory) markers (iNOS and CD86) stimulation with 200 ng/mL LPS for 4 hours. (E) mRNA levels of M2 (anti-inflammatory) markers (Arg-1 and CD163) stimulation with 200 ng/mL LPS for 4 hours. The following analytical methods were uniformly applied to panels A–E. The relative expression of each gene was calculated using the 2^−ΔΔCt^ method and normalized to GAPDH expression levels. One-way analysis of variance (ANOVA) followed by Dunnett’s multiple comparisons test was used for statistical analysis. All experiments were performed with three independent biological replicates (n = 3). ns, not significant, *p < 0.05, **p < 0.01, ***p < 0.001, ****p < 0.0001.

Given the role of neuroinflammation in neurodegeneration, we investigated whether Ataxin-3 regulates inflammatory responses and microglial polarization (Matos et al., 2011). CFTR acts as an anti-inflammatory factor. In immune cells, CFTR can modulate the activity of canonical inflammatory signaling pathways such as NF-κB. Loss of CFTR function leads to hyperactivation of the NF-κB pathway, thereby promoting the release of pro-inflammatory cytokines (Mullen et al., 2025). In our experiments, we overexpressed wild-type and mutant Ataxin-3 as well as knocked down Ataxin-3 in HMC-3 cells. By detecting the mRNA level of CFTR, we found that Ataxin-3 could downregulate the expression of CFTR at the transcriptional level (Figure 4A).

We next determined the mRNA levels of inflammatory cytokines (IL-6, IL-1β, and TNF-α) under basal condition (without LPS stimulation) and upon LPS treatment. We transfected HMC-3 cells with overexpressed Ataxin-3Q22, Ataxin-3Q80, or si-Ataxin-3-843, and induced inflammation by treating them with 200 ng/mL LPS for 4 hours (Supplementary Figure 4). qRT-PCR analysis revealed that overexpression of either wild-type or mutant Ataxin-3 elevates inflammatory responses under basal (LPS-unstimulated) conditions, and these inflammatory responses are further enhanced upon LPS stimulation. Specifically, both Ataxin-3Q22 and Ataxin-3Q80 significantly upregulated the mRNA levels of pro-inflammatory cytokines including IL-1β, IL-6 and TNF-α following LPS treatment. Interestingly, Ataxin-3Q80 induced a greater increase in IL-6 levels compared to the wild-type protein (Figure 4C).

We next examined microglial polarization. Overexpression of Ataxin-3Q22 and Ataxin3Q80 markedly elevated the LPS-induced expression of pro-inflammatory markers iNOS (2.8-fold and 3.4-fold, respectively) and CD86 (1.8-fold and 2.8-fold, respectively). The enhancement of pro-inflammatory marker levels mediated by Ataxin-3 overexpression was not affected by treatment with si-Ataxin-3-843 in response to LPS-induced inflammation, which is in contrast to the effects of Ataxin-3 overexpression (Figure 4D). In contrast, they downregulated the anti-inflammatory markers Arg-1 (to 50% and 28%, respectively) and CD163 (to 50% and 48%, respectively). Similarly, knockdown of Ataxin-3 by si-Ataxin-3-843 did not affect the anti-inflammatory response following LPS treatment (Figure 4E). These data indicate that Ataxin-3 promotes microglial polarization toward a pro-inflammatory phenotype, and the Ataxin-3 Q80 mutant drives this pro-/anti-inflammatory shift more potently than the wild-type protein.

## Discussion

This study elucidates a novel regulatory mechanism in microglia, demonstrating that the deubiquitinase Ataxin-3 regulates CFTR expression at both the mRNA and protein levels. At the protein level, Ataxin-3 paradoxically promotes CFTR degradation by modulating its K63-linked polyubiquitination. Although we cannot fully exclude the contribution of reduced CFTR mRNA levels, the Ataxin-3-mediated decrease in CFTR protein is functionally associated with microglial pro-inflammatory activation and promotes microglial polarization toward a pro-inflammatory phenotype. In the experimental system employed in this study, overexpression of either wild-type or polyQ-expanded mutant Ataxin-3 upregulated the expression of inflammatory factors under basal (LPS-unstimulated) conditions. Specifically, compared with the empty vector control, Ataxin-3Q22 and Ataxin-3Q80 increased the relative mRNA levels of IL-6 by 31.91% and 26.20%, those of IL-1β by 24.85% and 33.82%, and those of TNF-α by 31.95% and 39.94%, respectively. This observation is likely attributable to the basal in vitro culture conditions, which fail to recapitulate the pathological environment of chronic neurodegenerative progression or intense inflammatory stimuli. Previous studies have indicated that functional divergence between these two Ataxin-3 isoforms is typically more pronounced under such conditions (Araujo et al., 2011; Schuster et al., 2022). Nevertheless, the experimental conclusions of this study are sufficiently rigorous and compelling: we have clearly verified that Ataxin-3 regulates the activation status of microglia by modulating CFTR protein stability. This finding provides a novel research perspective and theoretical basis for elucidating the neuroinflammatory mechanisms underlying neurodegenerative diseases such as Machado-Joseph disease/spinocerebellar ataxia type 3 (MJD/SCA3).

Accumulating evidence has established that Ataxin-3 plays a key role in the transcriptional regulatory network by modulating the ubiquitination modification and stability of transcription factors. Specifically, Ataxin-3 can bind to the promoter regions of target genes or interact with transcription factors to regulate their transcriptional activity. Ataxin-3 is also capable of specifically binding to the genomic regions of target genes to mediate transcriptional regulation. Combined with our finding that Ataxin-3 can downregulate CFTR transcription, these data strongly suggest that Ataxin-3 may regulate CFTR expression in microglia through both transcriptional and post-translational mechanisms. The crosstalk between these two regulations may synergistically fine-tune CFTR protein levels, thereby amplifying the impact of Ataxin-3 on microglial inflammatory responses (Araujo et al., 2011; Evert et al., 2006; Wei et al., 2025).

CFTR, traditionally known as a chloride channel, is increasingly recognized for its role in inflammation (Mullen et al., 2025). Its dysfunction disrupts chloride ion homeostasis, which can potentiate the NF-κB pathway and trigger pro-inflammatory cytokine release (Chen et al., 2022; Mullen et al., 2025; Zhong & Pittman, 2006). Our data demonstrate that in microglia, Ataxin-3-mediated K63-linked deubiquitination targets CFTR for degradation. We propose that this loss of CFTR function releases a constitutive brake on inflammatory signaling cascades, thereby facilitating microglial activation and pro-inflammatory polarization.

It is particularly noteworthy that K63-linked ubiquitination generally serves non-proteolytic functions. Wild-type ubiquitin overexpression may saturate the cellular pool of K48-linked ubiquitin ligases that target CFTR for proteasomal degradation. This saturation would reduce the polyubiquitination of endogenous CFTR, thereby, thereby attenuating its recognition and clearance by the proteasome machinery. For ubiquitin variants that preferentially assemble non-degradative chains (e.g., K63-linked ubiquitin), overexpression could shift the balance of CFTR ubiquitination toward a protective, non-degradative modification (Evers et al., 2014). Our data thus imply an unconventional degradation pathway for CFTR, where K63 linkages may serve as a precursor or signal for secondary modifications. Notably, this degradation process is isoform-specific and ultimately enables Ataxin-3 to promote CFTR degradation via a dual degradation pathway: overexpressed Ataxin-3 facilitates CFTR degradation through the proteasomal and autophagic pathways, whereas mutant Ataxin-3 accelerates CFTR degradation via the proteasomal and lysosomal pathways, which is consistent with our inhibitor experiments (Figure 2D). These results also suggest that the effect involves protein degradation rather than reduced protein synthesis.

As core pathways of intracellular protein quality control, the proteasomal pathway and autophagic pathway precisely interact with each other to jointly maintain protein homeostasis, and they play a crucial role in the coordinated division of labor particularly in the degradation regulation of membrane proteins (*e.g.*, CFTR). These two pathways enable the precise selection of substrate degradation routes through distinct types of ubiquitin chains, while also engage in mutual cross-regulation. In addition, membrane-localized proteasomes can directly participate in maintaining the homeostasis maintenance of membrane-associated proteomes, thus forming a functional complement to the autophagic pathway. Specifically for the membrane protein CFTR, the proteasomal pathway serves as the central route in endoplasmic reticulum-associated degradation (ERAD), which is responsible for clearing misfolded CFTR; in contrast, autophagy exerts a compensatory degradation effect when the proteasomal function is overloaded or under specific physiological conditions (*e.g.*, membrane compartmentalization disorders during cellular aging). A similar division of labor has been observed from other membrane proteins (*e.g.*, mitochondrial membrane proteins): the proteasomal pathway preferentially degrades outer membrane proteins, whereas autophagy targets inner membrane and matrix proteins, thereby jointly ensuring membrane protein homeostasis and normal cellular function (Liebl & Hoppe, 2016; Prokisch & Büttner, 2024; Yoshii et al., 2011). Furthermore, we observed a cell-specific regulatory phenomenon: K63-linked chains promoted the degradation of Ataxin-3 itself in microglia, an effect was not detected in HEK293 cells. While the core focus of this study is the Ataxin-3-CFTR axis, the cell-specific regulation of Ataxin3 stability represents a compelling direction for future research. Given that microglia are key mediators of neuroinflammation in SCA3, this phenomenon may be closely related to the dysregulation of pathogenic protein homeostasis in SCA3.

Our research expands the functional repertoire of Ataxin-3 from neurons to immune cells and identifies a novel “Ataxin-3–K63 Ubiquitination–CFTR” axis with significant implications for neuroinflammation. While previous studies focused on the role of Ataxin-3 in neuronal protein homeostasis and autophagy (Nascimento-Ferreira et al., 2011; Paulino & Nóbrega, 2023), our work positions Ataxin-3 as a key regulator of microglial polarization, thereby revealing a previously unrecognized immune-regulatory function of this protein in neurodegenerative disease.

## Conclusions

In conclusion, we elucidate a mechanism whereby Ataxin-3 promotes CFTR degradation via modulating its K63-linked ubiquitination to drives pro-inflammatory activation of microglia. These findings provide a fresh perspective on SCA3 pathogenesis by linking Ataxin-3 dysfunction to microglia-mediated neuroinflammation, and identify potential therapeutic target for SCA3.

## Supplementary Material

Supplementary Figure2.png

Supplementary Figure4.png

Supplementary Table1.pdf

Supplementary Table2.pdf

Supplementary Figure3.png

Supplementary Figure1.png

## Data Availability

All data generated or analyzed during this study are included in this published article and its supplementary information files.
