# Supplementary figures and images for: Polyglutamine-Expanded Ataxin-3 Accelerates CFTR Degradation Through K63-Linked Ubiquitination to Exacerbate Microglial Inflammation
Source: ASN Neuro. 2026 May 26;18(1):2662867. doi: 10.1080/17590914.2026.2662867 (PMC13215304; doi:10.1080/17590914.2026.2662867)

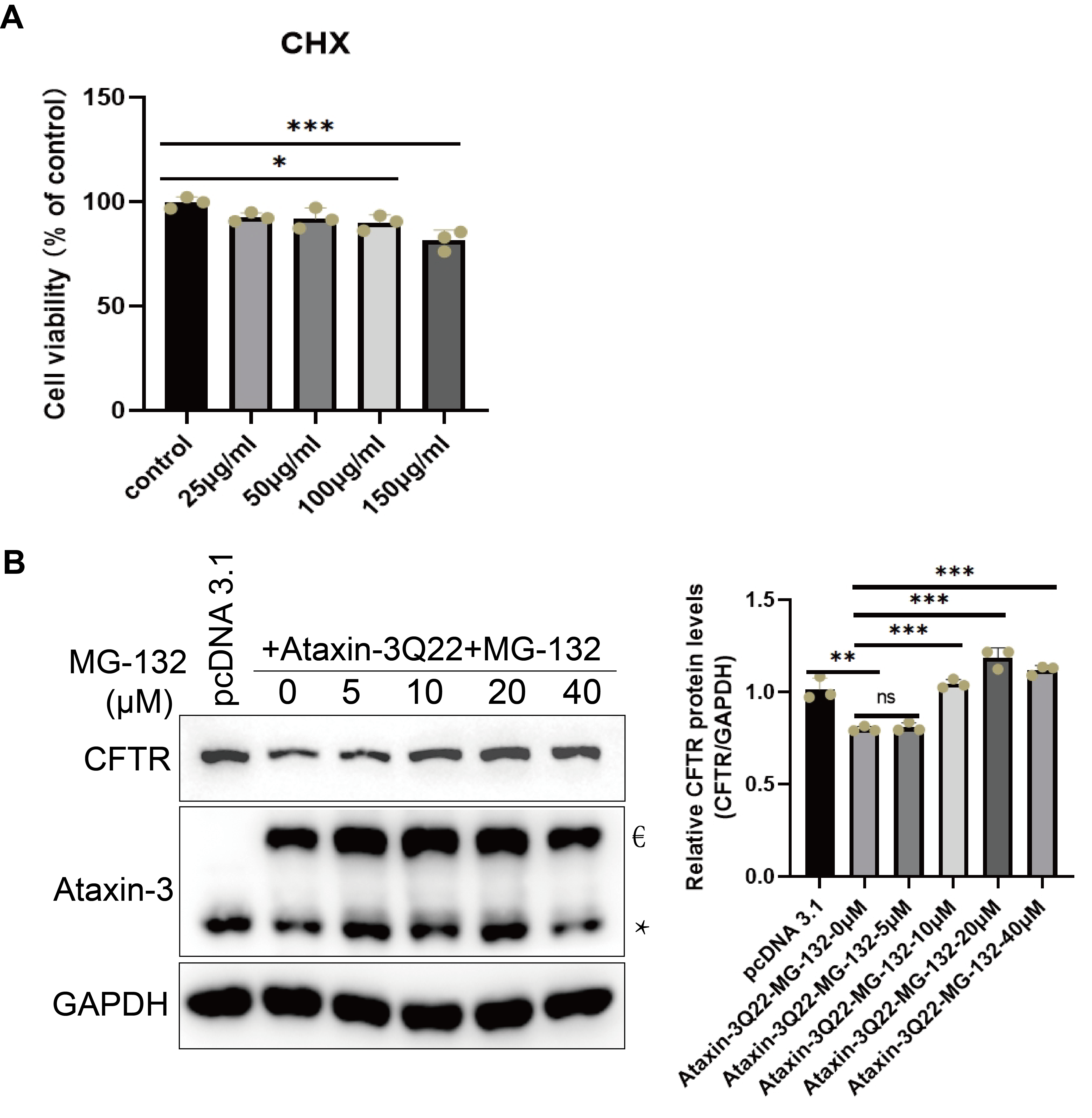

Supplement: Supplementary Figure2.png [file TASN_A_2662867_SM6806.png]

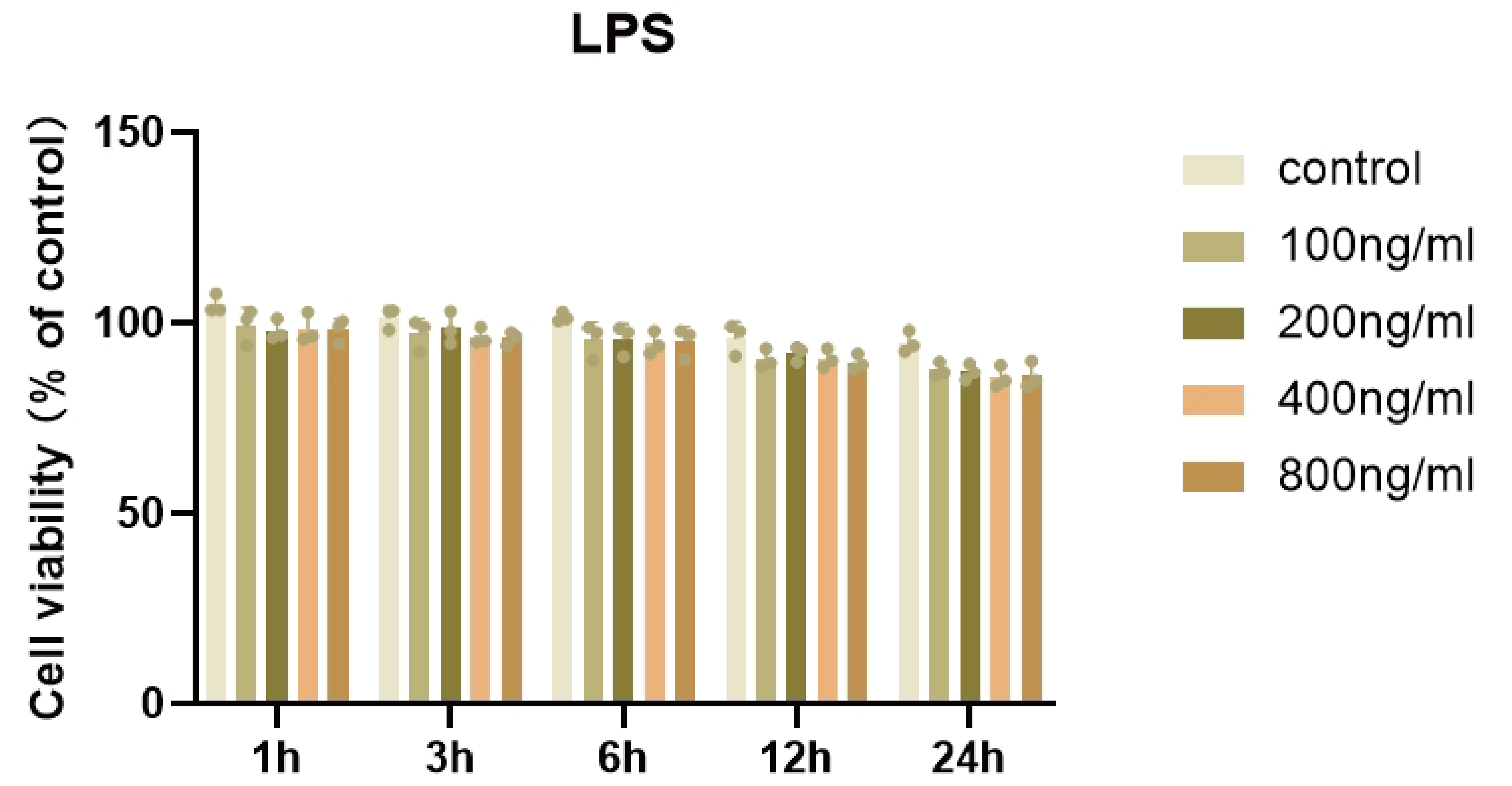

Supplement: Supplementary Figure4.png [file TASN_A_2662867_SM6805.png]

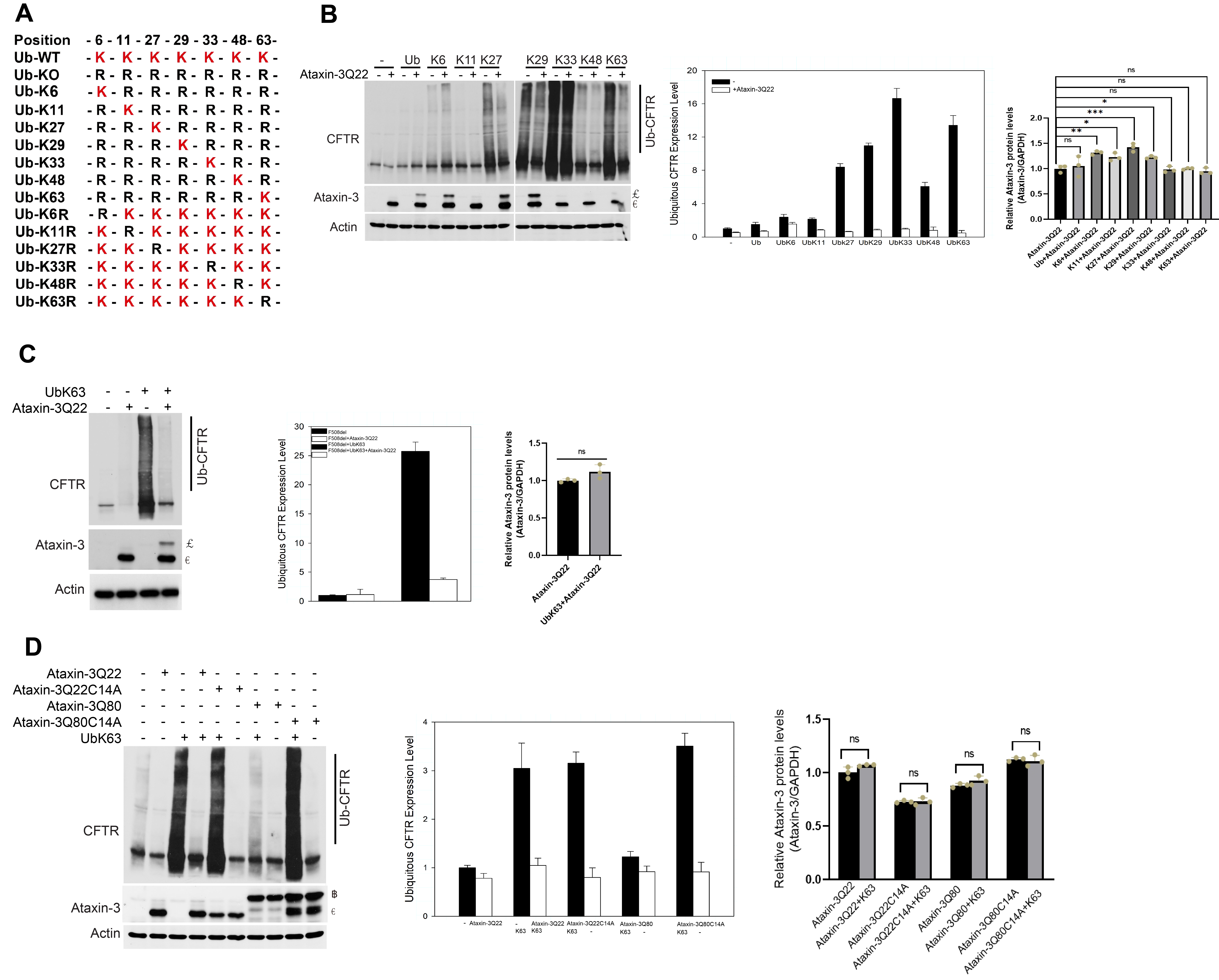

Supplement: Supplementary Figure3.png [file TASN_A_2662867_SM6802.png]

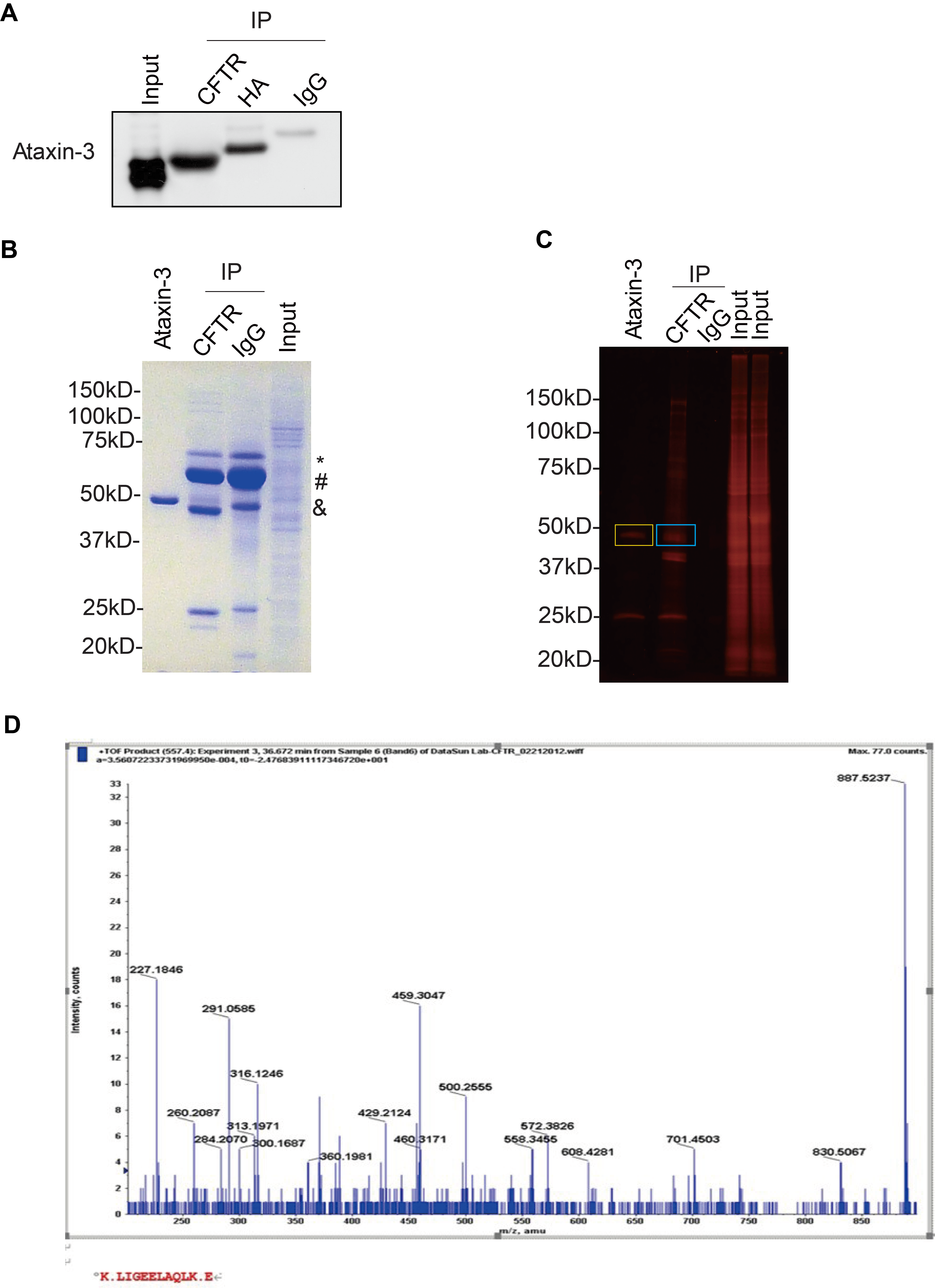

Supplement: Supplementary Figure1.png [file TASN_A_2662867_SM6801.png]
