# Supplementary material for: Polyglutamine-Expanded Ataxin-3 Accelerates CFTR Degradation Through K63-Linked Ubiquitination to Exacerbate Microglial Inflammation
Source: ASN Neuro. 2026 May 26;18(1):2662867. doi: 10.1080/17590914.2026.2662867 (PMC13215304; doi:10.1080/17590914.2026.2662867)
Supplement: Supplementary Table1.pdf [file TASN_A_2662867_SM6804.pdf]

Supplementary Table 1: siRNA used in this study.

Target genes	Sequences(5’-3’)
Ataxin-3siRNA#1	GAGUUACUAGUGAAGAUUA
Ataxin-3siRNA#2	GAUCGAUCCUAUAAAUGAA
Ataxin-3siRNA#3	GGAAGAGACGAGAAGCCUA
