# Supplementary material for: Polyglutamine-Expanded Ataxin-3 Accelerates CFTR Degradation Through K63-Linked Ubiquitination to Exacerbate Microglial Inflammation
Source: ASN Neuro. 2026 May 26;18(1):2662867. doi: 10.1080/17590914.2026.2662867 (PMC13215304; doi:10.1080/17590914.2026.2662867)
Supplement: Supplementary Table2.pdf [file TASN_A_2662867_SM6803.pdf]

Supplementary Table 2：PCR primers used in this study

Name qRT-PCR	Sequences(5'-3')
CFTR-F(human)	GGTTGTGCTGTGGCTCCTTG
CFTR-R(human)	ATACGAACTGGTGCTGGTGATAATC
IL-6-F(human)	AGTGAGGAACAAGCCAGAGC
IL-6-R(human)	GGTCAGGGGTGGTTATTGCA
IL-1β-F(human)	CCACCTCCAGGGACAGGATA
IL-1β-R(human)	TCAACACGCAGGACAGGTAC
TNF-α-F(human)	CTTCCAGCTGGAGAAGGGTG
TNF-α-R(human)	CCCAAAGTAGACCTGCCCAG
GAPDH-F(human)	GTCAAGGCTGAGAACGGGAA
GAPDH-R(human)	AAATGAGCCCCAGCCTTCTC
iNOS-F(human)	ATGGGAGAAGGGGATGAGCT
iNOS-R(human)	GTCCCAGGTCACATTGGAGG
CD86-F(human)	GGAAGAAGAAGAAGCGGCCT
CD86-R(human)	CGCTGGGCTTCATCAGATCT
Arg-1-F(human)	AAGATTCCCGATGTGCCAGG
Arg-1-R(human)	GTCCACGTCTCTCAAGCCAA
CD163-F(human)	GCGGGAGAGTGGAAGTGAAA
CD163-R(human)	ACCTGCACTGGAATTAGCCC
